# Effect of sinusoidal injection velocity on miscible viscous fingering of a finite sample: Nonlinear simulation

**DOI:** 10.1016/j.heliyon.2023.e14480

**Published:** 2023-03-15

**Authors:** Syed Zahid, Surfarazhussain S. Halkarni, Tapan Kumar Hota

**Affiliations:** a Department of Mechanical Engineering SRM University-AP Andhra Pradesh 522 240, India; b Department of Mathematics SRM University-AP Andhra Pradesh 522 240, India

**Keywords:** Viscous fingering, Time-dependent injection, Porous media

## Abstract

The effect of a sinusoidal injection on the fingering instability in a miscible displacement in the application of liquid chromatography, pollutant contamination in aquifers, etc., is investigated. The injection velocity, U(t) is characterized by its amplitude of Γ and time-period of T. The solute transport, flow in porous media, and mass conservation in a two-dimensional porous media is modeled by the convection-diffusion equation, Darcy's equation, and the continuity equation, respectively. The numerical simulation is performed in COMSOL Multiphysics utilizing a finite-element based approach. The fingering dynamics for various time-period have been studied for two scenarios namely, injection-extraction (Γ>1) and extraction-injection (Γ<−1). The onset of fingers and vigorous mixing is observed for Γ>1, whereas for Γ<−1, the onset gets delayed. The viscosity contrast between the sample and the surrounding fluid is characterized by the log-mobility ratio R. When R>0 the rare interface becomes unstable, while for R<0 the frontal interface deformed. In the case of R<0, the extraction-injection process attenuates the fingering dynamics, which is beneficial in chromatographic separations or pollutant dispersion in underground aquifers. The injection-extraction process is observed to have a longer mixing length, indicating early interaction between both interfaces. The degree of mixing χ(t) is more pronounced for injection-extraction scenario and least for extraction-injection R<0,Γ=−2. The average convective forces are more dominant for Γ>1,R=2 till the deformed rare interface interact with diffusive frontal interface. The average diffusive forces are significant for Γ<−1,R=−2 which can be helpful in separation of chemicals in chromatography. This study therefore provided new insights into the role of alternate injection-extraction injections in altering the fingering dynamics of the miscible sample.

## 1 Introduction

In porous media, flow instabilities can be observed when a high-viscosity fluid is displaced by a low-viscosity fluid. This instability, which manifests as finger-like patterns, is encountered frequently in numerous engineering and environmental applications. This instability, which manifests itself as finger-like patterns, is commonly known as viscous fingering (VF) [1]. Viscous fingering is particularly important in the disciplines of hydrogeology, chemical separation, and oil recovery, to name a few [1,2]. For instance, in the case of enhanced oil recovery, VF occurs for both miscible and immiscible fluids when crude oil is displaced [1]. The unfavourable viscosity contrast between injected solvent and oil in miscible displacements encountered in enhanced oil recovery processes usually results in viscous fingering, which results in lower sweep efficiency and oil recovery. To comprehend the VF process, numerous theoretical and experimental studies have been carried out [1,2]. The fundamental cause of the instability at the interface is typically attributed to the viscosity and/or density contrast [1]. To better understand how fingering instability occurs, various geometrical configurations have been used, including rectilinear [1,3], radial [4,5], and quarter-five-spot [6] to name a few.

However, if the less viscous region is localized within a very narrow region, as is common in contaminant spreading in groundwater and sample dispersion in a chromatographic column, the stability characteristics are significantly different than in the case of a semi-infinite extent [7]. It is common for a localized or finitely extended water-soluble contaminant to be surrounded by groundwater whenever a contaminant spreads into the groundwater system. If the viscosities of the contaminant and water are dissimilar, miscible viscous fingering may occur and affect the spreadability of the contaminant. Further, VF contributes to the enlargement of the sample and the acceleration of its spreading when a finite amount of a more viscous fluid is surrounded and displaced by a less viscous miscible fluid. Using the quasi-steady-state approximation Rousseaux et al. [8] investigated the viscous fingering phenomena in a chromatographic column and reported the effect of finite sample size. Further, it is noted that the impact of displacement velocity and mobility ratio on the beginning and progression of viscous fingers. In column chromatography, a carrier fluid displaces a more or less viscous, finite-width sample in a porous medium [7]. De Wit et al. [7] studied the VF of a more viscous sample that was displaced by a less viscous fluid. They observed that, unlike a single interface, as the viscosity decreases with time, the VF of a finite slice is a transient phenomenon. Viscous fingering leads to distortion of the sample and broadening of the peaks. The widening of the peaks led to an increase in variance. This reveals that the dynamics of fingering are influenced by the presence of a finite amount of fluid and that VF with a double interface has distinct features from VF with a single interface. Further, Mishra et al. [3] concluded that the less viscous sample had a greater variance than the more viscous sample. The emergence of fingering instability at the frontal or rear interface was also observed in experimental liquid chromatography experiments by Mayfield et al. [9]when a finite solute plug of higher or lower viscosity was displaced by a less viscous solvent. A comprehensive analysis of VF in chromatographic columns was examined by Rousseaux et al. [8]. Additionally, Zhou et al. [10] carried out experiments to investigate the mechanisms of chemical flooding for enhanced oil recovery. They experimented with one chemical slug and two chemical slug injections in a 2D sand-packed cell and discovered that the two chemical slug injections can recover more oil if a water slug injection is used in between the two chemical slugs. In order to understand how VF in radial displacement differs from that in rectilinear displacement, a theoretical and experimental analysis is reported by Sharma et al. [11] for a miscible annular ring in radial geometry. They have found that the VF of a miscible annular ring is a persistent phenomenon as opposed to the transient nature of the VF of a miscible slice. In contrast to rectilinear flow, they discovered that the variance in the annular ring is a non-monotonic function of time.

In order to control, recently, the time-dependent injection strategy is used. The time-dependent injection velocity varies with time, resulting in different flow dynamics and displacement instabilities. In trickle-bed reactors, which include three stages of injection, soaking, and production, this phenomenon is also observed. In EOR, for example, the cyclic steam stimulation (CSS) technique is utilized [12]. The recovery of heavy crude oil involves solvent-based Cyclic Solvent Injection (CSI) similar to CSS [13]. In case of single interface, Chen and Meiburg [6] investigated flows in a quarter five-spot geometry with a time-dependent injection rate. These authors have found that initially lower injection rates can partially stabilize the flows, while larger ones at the later stages have an almost negligible effect on the recovery at breakthrough. Further, and Elgahawy and Azeiz [14] reported time-dependent injection rates based on a sinusoidal velocity model for miscible VF. They observed that, based on different combinations of time period (T) and amplitude (Γ), their sinusoidal velocity model can be more or less unstable than the constant injection scenario. The larger periods in the sinusoidal model resulted in enhanced instability with more developed fingers for (Γ<−1), whereas (Γ>1) results in a more diffusive front with fewer fingers, weakening the instability. In another study, Yuan and Azeiz [15] utilized periodic displacement cycles that involved alternating stages of injection and production or injection and soaking. These authors observed that the time period and amplitude of the flow velocity, along with the approach of displacement initiation, had a drastic impact on the flow dynamics. They observed that depending on the cycle period, amplitude, and displacement circumstances, altering displacement rate can either stabilize or destabilize VF. Furthermore, Yuan and Azeiz [16] reported that the cycle period and velocity amplitude, which are heavily influenced by the inertia of the fluid, had a significant impact on the stability. In immiscible displacement in EOR, a number of experimental studies on the flow instability of time-dependent displacement flows were conducted. In addition, it is observed that this periodic injection could delay the breakthrough and boost oil sweep efficiency and recovery. For instance, in oil field-wide stimulation trials, Spanos et al. [17] demonstrated that the pressure pulsing method with the proper period and amplitude can significantly increase production in heavy and light oil deposits. Depending on whether the velocity model is injection-soak or vice versa. Despite the vast number of studies on viscous fingering in a finite slug with constant injection velocity. To the best of the authors' knowledge, the effect of time-dependent velocity, particularly in a cyclic injection-extraction manner, has not been explored for finite sample.

The current study looked at the effects of a simple cyclic time-dependent displacement rate on the onset and progression of VF in a miscible slice. It is expected that depending on the cycle period, amplitude, and displacement scenarios, such varying displacement rate can either stabilize or destabilize VF. The most important mechanism is that such time-dependent rates can effectively alter the competition between convection (destabilizing effect) and dispersion (stabilizing effect). This contrasts with the commonly used constant injection rate where the flow instability is determined by the Peclet number and mobility contrast for a given scenario. This motivates us to understand the effect of sinusoidal time-dependent displacements on the miscible finite sample with the following objectives.

(i) to compare and analyze the resemblance and contrast between the constant displacements and time-dependent displacements for a miscible finite sample.
(ii) to study the effect of injection-extraction (Γ>1) and extraction-injection (Γ<−1) processes on the fingering dynamics of a finite sample.
(iii) to understand the impact of increasing time-period on the VF of the sample.

The quantitative analysis of fingering instability is derived from the concentration field and the statistical analysis of variance. Further, the interfacial length, degree of mixing, mixing length, and interaction of diffusive and convective forces are discussed in addition to assessing the degree of instability and capturing the development of the fingers. The structure of the paper is organized as follows: The mathematical formulation and physical problem are presented in section 2, followed by numerical simulation using COMSOL Multiphysics in section 3. The results obtained are discussed in section 4 and the conclusions are drawn in section 5.

## 2 Mathematical formulation

### 2.1 Physical description of the model

**Fig. 1:**
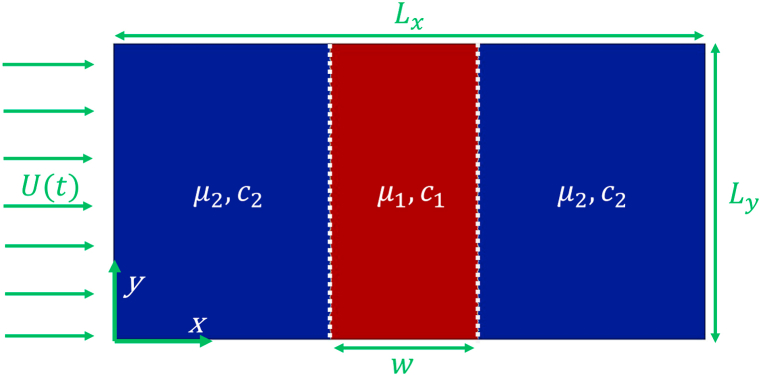
Schematic of the flow configuration in a Hele-Shaw cell (or a 2D homogeneous porous media) where a sample of width w surrounded by another fluid (top view). The dotted lines represent the initial unperturbed interfaces.

Consider a cyclic rectilinear displacement of a viscous fluid at speed U(t) [Eq. (1)] displacing a miscible, neutrally buoyant, fluid sample of width w in a Hele-Shaw cell (consisting of two parallel plates $L_{x}$ and $L_{y}$, separated by a small gap, $b\ll L_{y}$) as shown in Fig. 1. Moreover, we assume that the two fluids are Newtonian, incompressible, fully miscible, and non-reactive. Darcy's law [Eq. (3)], which is mathematically analogous to the flow in a homogeneous porous medium of constant permeability $\kappa =\frac{b^{2}}{12}$, describes momentum conservation in a Hele-Shaw cell. The displacing and sample fluid viscosities are $\mu_{1}$ and $\mu_{2}$, respectively, and depend on the solute concentration c. A convection-diffusion equation is used to calculate the evolution of solute concentration [Eq. (4)]. Further, the inertia effect is not included in our analysis. It must be noted that Darcy's equation is actually not valid in cases where inertia plays a relevant role, such as for Hele-Shaw cells with a large gap, low viscosity fluids, or large displacing velocities [18].

### 2.2 Sinusoidal time-dependent injection velocity

**Fig. 2:**
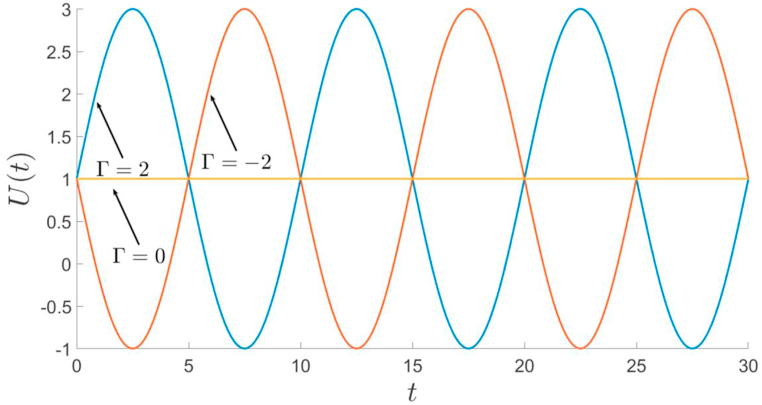
The time-dependent sinusoidal injection velocity, $U\left(t\right)=U_{0}\;\left(1+\Gamma \;\sin\left(2\;\pi \;t/T\right)\right)$ for T=10, Γ=−2,0,2 and $U_{0}=1$.

Following [14], the injection velocity is considered as a cyclic one and given by

(1) $$U\left(t\right)=U_{0}\left[1+\Gamma \;\sin\left(2\pi t/T\right)\right]$$

where T represents time period and Γ is the amplitude. The constant velocity formulation can be recovered by substituting Γ=0 [1,18]. Note that when |Γ|≤1, the displacement rate is positive and the flow undergoes simple injection, whereas when |Γ|>1, the flow alternates between injection $\left(U\left(t\right)>U_{0}\right)$ and extraction $\left(U\left(t\right)<U_{0}\right)\text{.}$ Hence the displacement will be triggered by extraction when Γ<1 and by injection when Γ>1. Therefore, Γ<1 corresponds to an extraction-injection process while Γ>1 indicates an injection-extraction process. For the values of Γ=−2,0,2 and time period T=10, the typical profiles of sinusoidal displacements are represented in Fig. 2. It can be observed that in extraction process U(t) can be negative.

### 2.3 Governing equations

It is a general practice to present the governing equations in a moving frame of reference [7,14,18]. In the present work, the analysis is presented in a stationary frame of reference. Thus, the flow in a homogeneous porous media with cyclic injection is governed by Refs. [1,14].

(2) $$\nabla ∙\vec{u}=0$$

(3) $$\nabla P=-\frac{\mu }{\kappa }\;\vec{u}$$

(4) $$\frac{\partial c}{\partial t}+\vec{u}∙\left(\nabla c\right)=\nabla \cdot \left(D\nabla c\right)\text{,}$$

where $\vec{u}=\left(u,v\right),p$, and c are Darcy's velocity, pressure, and concentration of the solute, respectively. The permeability κ and the molecular diffusivity D are constants in the present study. Further, the continuity equation, Eq. (2) is due to incompressible assumption. The physical model discussed abides by two interesting physical phenomena: (a) hydrodynamics, which represents fluid flow in porous media, and (b) solute transport in porous media. Following the previous works, an Arrhenius-type relationship between the viscosity and solute concentration is assumed which is shown in Eq. (5) as [1,7]:

(5) μ(c)=μ1eRcc2

where the log-mobility ratio is defined as $R=\ln\left(\mu_{2}/\mu_{1}\right)$. In the case of R<0 indicates $\mu_{2}<\mu_{1}$; the finite sample becomes less viscous than the displacing fluid, and the instabilities occur at the frontal interface while the rear interface remains stable. When R>0, then $\mu_{2}>\mu_{1}$ and the finite sample is more viscous than the carrier fluid, the rear interface becomes unstable with fingers being formed while the frontal interface is stable. To complete the mathematical formulation the boundary conditions for velocity and concentration needs to be mentioned. The boundary conditions for the velocity vector given in Eqs. (6), (7) are:

(6) $$\vec{u}=\left(U\left(t\right),0\right)\;at\;x=0$$

(7) $$\frac{\partial u}{\partial x}=v=0\;at\;y=0\;and\;y=L_{y}$$

For the case of species concentration, to ensure that there is no diffusive flux across the boundary, we have used the Neumann boundary condition. The boundary condition for pressure is shown in Eq. (8)

(8) $$p=0\;at\;x=L_{x}$$

## 3 Non-linear simulations in COMSOL Multiphysics®

To investigate and analyze the non-linear evolution of the fingers and to quantify the various stability factors the governing equations (2), (3), (4), (5) have been incorporated in COMSOL Multiphysics 5.3a [19]. In our study, the two different physics interfaces of finite element method-based COMSOL Multiphysics 5.3a have been used to model the hydrodynamics and solute transport. The hydrodynamic part is modeled using Darcy's law (dl) model given by:

(9) $$\frac{\partial }{\partial t}\left(\varepsilon_{p}\rho \right)+\nabla ∙\left(\rho u\right)=Q_{m}$$

(10) $$u=-\frac{\kappa }{\mu }\nabla p$$

For constant porosity $\varepsilon_{p}$, permeability κ and fluid density ρ and safely disregarding the mass source $Q_{m}$, Eqs. (9), (10) reduces to the continuity equation, Eq. (2) and Darcy's law Eq. (3), respectively. The transport of solute concentration is modeled by utilizing the transport of diluted species in porous media (tds) model whose equations are as follows:

(11) $$P_{1,i}\frac{\partial c_{i}}{\partial t}+P_{2,i}+\nabla ∙\Gamma_{i}+u∙\nabla c_{i}=R_{i}+S_{i}\text{,}$$

with

$$P_{1,i}=\left(\varepsilon_{p}+\rho k_{p,i}\right),N_{i}=\Gamma_{i}+uc_{i}=-D_{e,i}\nabla c_{i}+uc_{i}D_{e,i}=\frac{\varepsilon_{p}}{\tau_{F,i}}D_{F,i}$$

Here $R_{i},c_{i},S_{i},D_{e,i},D_{F,i},\Gamma_{i},\tau_{F,i}$ denotes the reaction rate, concentration, source, effective diffusion, molecular diffusion, diffusive flux, and tortuosity of the i-th species (i=1,2) respectively [19]. Assuming porosity and diffusion to be constant and employing the tortuosity model which gives $\tau_{F,i}=1$, the system of equations from Eq. (11) reduces to the convection-diffusion equation, Eq. (4).

To solve the hydrodynamics and the transportation of solutes, at the transverse boundaries no-flow boundary condition is specified. At the inlet, a normal inflow velocity is prescribed, while at the outlet the pressure is zero. In the case of species concentration, the inflow boundary condition has c=0, and outflow corresponds to free flow. For the concentration the initial condition is specified as shown in Eq. (12):

(12) $$c\left(x,y,t=0\right)=\frac{c_{2}}{2}\left[\mathrm{erf}\left(\frac{x-x_{0}}{\delta }\right)-\mathrm{erf}\left(\frac{x-x_{0}-w}{\delta }\right)\right]$$

where erf(⋅) represents the error function and the δ represents diffusive zone. The length $L_{x}$ and width $L_{y}$ for aquifers and chromatographic columns varies relatively over a wide range depending on system's geometry, flow velocity, and particle diameter [7,8]. The typical computational domain for this study constitutes an aspect ratio of 4 with length $L_{x}=2m$ and width $L_{y}=0.5m$ for R>0 and length $L_{x}=3m$ and width $L_{y}=0.75m$ for R<0. An aspect ratio of 4 was selected to have optimal fingering dynamics. The sample length w was taken to be $\frac{L_{x}}{8}$ which corresponds to 0.25m and 0.375m for R>0 and R<0. The rear interface of the sample was initially located at x=0.5m for both R>0 and R<0. The position of the finite sample can affect the fingering and hence the mathematical formulation as reported by Kumar and Mishra [20]. The details of various parameters used in the study are presented in Table 1.

**Table 1:** Summarizing the different parameters used in the study.

Parameters	Symbol	Value & Unit
Length of the domain	$L_{x}$	2, 3 m
Width of the domain	$L_{y}$	0.5, 0.75 m
Sample width	w	0.25, 0.375 m
Log-mobility ratio	R	2, −2
Viscosity of the displacing fluid	$\mu_{1}$	0.001 Pa S
Density of both the fluids	ρ	1000 kg/m^3^
Porosity	$\varepsilon_{p}$	0.5
Permeability	κ	10^−6^ m^2^
Diffusion coefficient	D	4 × 10^−8^ m^2^/s
Interface position	$x_{0}$	0.5 m
Thickness of the transition zone	δ	10^−5^ m

Before analyzing the results and presenting the stability quantification, it is necessary to discuss mesh refinement and address the numerical dispersion. It is widely reported that fingering dynamics are sensitive to mesh refinement, for details one can refer to the work of Rabbani et al. [21] and the reference their in. To analyze the effect of mesh resolution on displacement of a finite miscible slice, the simulations have been carried out using four different mesh sizes available in COMSOL, namely, fine, finer, extra fine, and extremely fine. It is observed that for the coarse mesh resolution, such as fine and finer mesh resolution, there is no instabilities were appearing due to poor grid resolution. Hence the numerical solution obtained using fine and finer mesh resolution are not consistent which is also reported by Rabbani et al. [21] and Sharma et al. [11]. Upon using the extra-fine and extremely-fine meshes, it is observed that the results are meticulously consistent. So, the extra-fine mesh has been used for the numerical simulations as it takes less computational time and memory space. For better clarity, a comparison of various parameters in using different mesh resolution is presented in Table 2. It is noted as one goes from coarser to finer mesh resolution, the computational time increases but the mean elemental size improved. The mean element size helps in understanding mesh resolution. As the mean element size is less in extra fine mesh in comparison to the other mesh size, the fingering dynamics is captured appropriately for the extra fine mesh. Further, the whole computational domain is discrteized using Delaunay triangulations. The advantage of using Delaunay triangulation over other existing unstructured meshes is that it creates a natural disturbance at the interface that remains the same for all simulations and it ensures that the fingering patterns don't change and stay independent of the mesh, and the triangles maintain their shape all over the domain [11,22]. This removes the additional use of random initial conditions to generate the perturbations at the interface. This was also reported by Bakharev et al. [23] and Sharma et al. [11] in a similar analysis of viscous fingering problems.

**Table 2:** Comparison of three different meshes.

Mesh type	Degrees of freedom	Computation time (s)	Mean Element Size
Fine	49,460	20,160	0.017
Finer	77,355	31,740	0.014
Extra fine	358,756	151,200	0.006

In order to remove any numerical dispersion in our numerical simulation, the consistent stabilization scheme in COMSOL has been used. The streamline and crosswind stabilization schemes are implemented as part of consistent stabilization. These stabilization schemes ensure that the obtained solution is more robust and computationally efficient. In particular, the streamline stabilization only adds diffusion in the direction of the flow, whereas the crosswind diffusion terms add both diffusive and discontinuous terms, which is helpful in the numerical overshoots and undershoots. This results in less numerical dispersion and makes sure that the numerical solutions are consistent. For the temporal discretization, the default backward difference formulation of first and second orders is employed. Following Sharma et al. [24], the initial and maximum time step is taken to be $10^{-2}$ so that the results remain independent of the final time. To solve the non-linear equations, the direct solver MUMPS is used, and the results are compared with another direct solver, PARDISO. It is observed that the later solver takes less time to converge the solution, but the time difference is not substantial. Further, the MUMPS solver has been used successfully in recent studies [11,24]. In the coming sections, the qualitative and quantitative behaviour of fingering dynamics is be presented.

## 4 Result and discussion

In this section, the onset and nonlinear dynamics of fingers is presented using the species concentration. Further, the statistical quantification is discussed to analyze the effect of fingers on the finite slice with respect to the time-dependent injection velocity, U(t).

### 4.1 Fingering profile

The effect of the injection-extraction (Γ>1) and extraction-injection (Γ<1) process on the fingering dynamics of a finite miscible slice is characterized. It may noted that we have two cases depending the viscosity contrast between the sample and surrounding fluid, that is R>0 (when sample is more viscous) and R<0 (when the sample is less viscous).

**Fig. 3:**
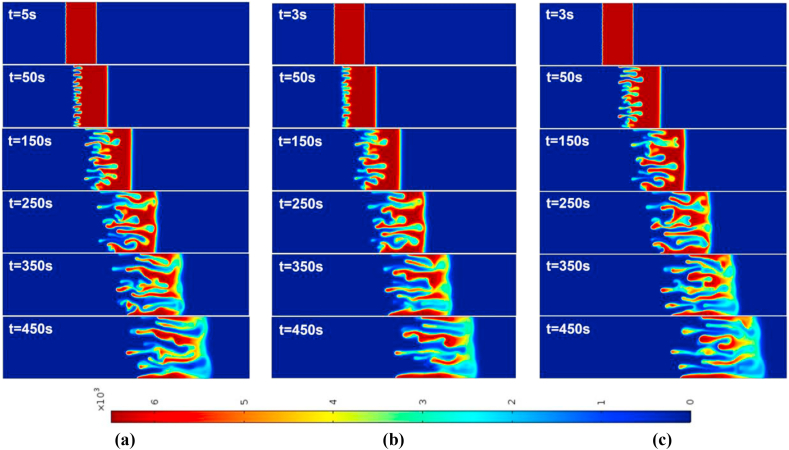
For Γ=2,R=2, the spatial evolution of species concentration for (a) constant injection U=0.001m/s (b) T=50 (c) T=100 at different times. The onset time of instability is slightly early in all time-dependent cases. The fingering dynamics remain the same at early times, later the dynamics differ with the fingers reaching the frontal interface after 250s.

**Fig. 4:**
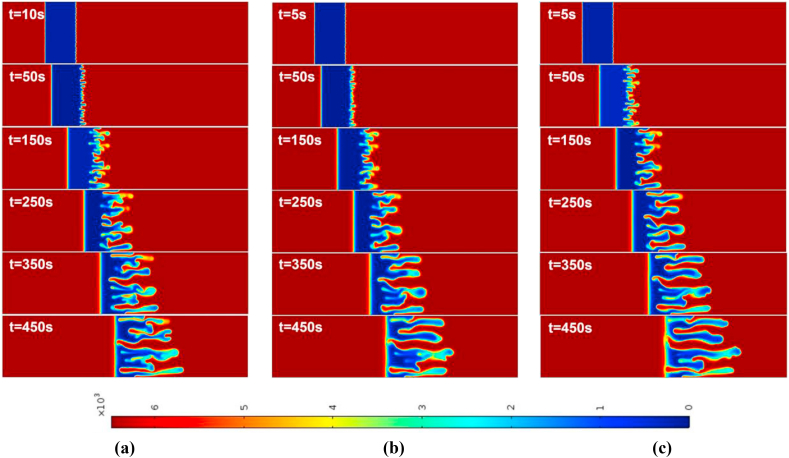
For Γ=2,R=−2 the spatial evolution of species concentration for (a) constant injection U=0.001m/s (b) T=50 (c) T=100 at different times. The onset time of instability is a bit early in all time-dependent cases. The fingering dynamics remain the same at early times, later the dynamics differ with the fingers reaching the rear interface after 450s.

For the injection-extraction case with Γ=2 and time-period T=50,100, the density plots are shown in Fig. 3, Fig. 4 for R>0 and R<0, respectively. As reported by Mishra et al. [3], the rear interface is deformed for R>0 and the frontal interface is deformed for R<0 and the finger like patters is observed. The fingers carrying the less viscous fluid move downstream, while the reverse fingers carrying the more viscous fluid move upstream. They are known as forward and backward fingers, respectively. At early time, till t=50s, the fingering dynamics in R>0 and R<0 is remain similar for respective values of T. After t=150s, the peculiarities associated with the finite sample size become apparent. Regarding VF with two interfaces, one unstable and one stable, begin to interact. In the event that R>0, the stable frontal zone acts as a barrier that prevents the forward fingers from propagating further downstream and forces them to reverse their orientation once the two fronts interact.

After t=150s, this sort of reverse displacement can be observed. For R<0, the stable zone at the rear interface acts as a barrier to the propagation of backward fingers in the opposite direction of the flow, favoring the growth of forward fingers after t=150s. During this transitional phase, the reorientation of the fingers improves the mixing of the sample [3]. This is discussed in Fig. 15 (a)-(d).

**Fig. 5:**
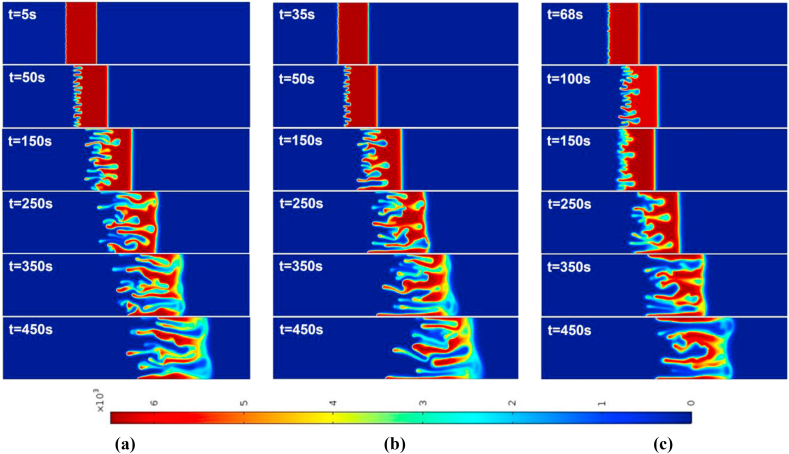
For Γ=−2,R=2 the spatial evolution of species concentration for (a) constant injection U=0.001m/s (b) T=50 (c) T=100 at different times. The onset of instability is delayed in all time-dependent cases. The fingering dynamics remain the same for initial times, later the dynamics differ with the fingers reaching the frontal interface after 250s. However, for T=100 the interface interaction occurs after 350s.

**Fig. 6:**
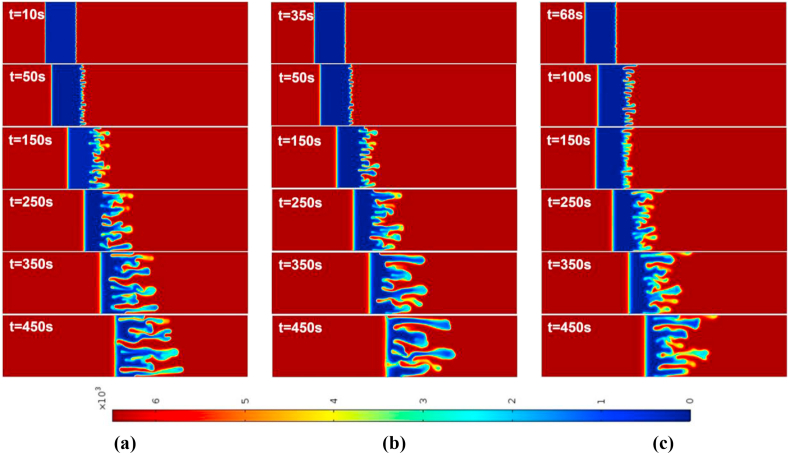
For Γ=−2,R=−2 the spatial evolution of species concentration for (a) constant injection U=0.001m/s (b) T=50 (c) T=100 for different times. The onset time of instability is monotonically increasing with increasing values of T for time-dependent cases. The fingering dynamics remain the same at early times, later the dynamics differ with the fingers reaching the rear interface after 450s.

For the case of an injection-extraction process, either for R>0 (see Fig. 3) or R<0 (see Fig. 4), the onset of instability is early for all time periods in contrast to constant injection. It is observed that with increase in T the formed fingers move faster and hence early interaction of the frontal and rear interfaces. In addition, at higher time periods less number of fingers are observed than that of constant injection case. For the extraction-injection case with Γ=−2 and time-period T=50,100, the temporal evolution of concentration plots are depicted in Fig. 5, Fig. 6 for R>0 and R<0, respectively. The onset of instability gets delayed than the constant injection flow. The onset is monotonically increasing with increasing value of T in contrast to injection-extraction case where the onset monotonically decreasing with increase in T. Such a delay can be attributed to the fact that in the case of Γ<1, the velocity is initially less than the mean in the first half cycle, causing diffusion to dominate over convection, resulting in the delay of the formation of fingers. It is important to understand the differences between the injection-extraction and extraction-injection processes.

For a given T and R the onset of instability gets delayed in extraction-injection (t=38s for T=50 and t=68s for T=100) than the injection-extraction (t=3s for T=50 and t=5s for T=100) scenario. The interaction of the stable and unstable interface occurs early for injection-extraction case (t∼250s), indicating prominent mixing, whereas for extraction-injection case the interfaces interaction occurs at t∼450s reflecting a reduced mixing. To explore the differences further in injection-extraction and extraction-injection processes, a quantitative analysis of the simulations as shown in Figs. 3–6 is analyzed in the next two subsections.

### 4.2 Average concentration profiles

Transversely averaged concentration profiles are a classical measure carried out in theoretical [7,8] and experimental studies of VF [25]. It provides insight into the spatial and temporal properties of the concentration and allows a better understanding of the properties of the dynamics averaged over the cross-sectional area [3]. The transversely averaged concentration is denoted by $c_{avg}$ and defined as represented by Eq. (13)

(13) $$c_{avg}\left(x,t\right)=\frac{1}{L_{y}}\;\int_{0}^{L_{y}}c\left(x,y,t\right)\;dy$$

**Fig. 7:**
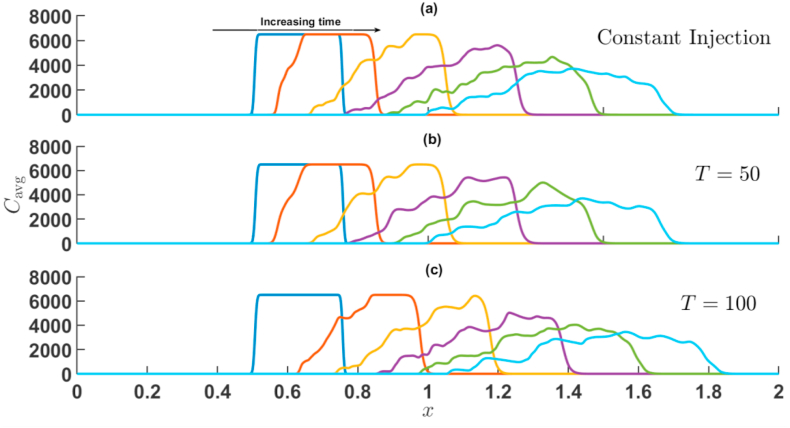
For Γ=2,R=2 the transversely averaged concentration profiles corresponding to the density plots shown in Fig. 3 at (a) constant injection U=0.001m/s (b) T=50 (c) T=100. The arrow shows the increase in time. The time stamps are same as in Fig. 3.

**Fig. 8:**
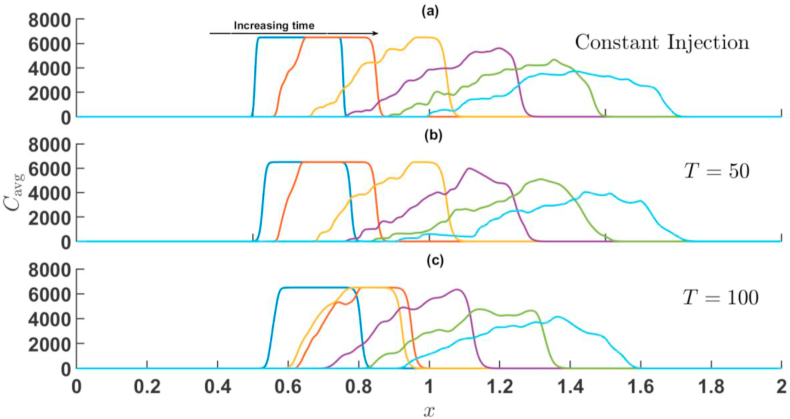
For Γ=−2,R=2 the transversely averaged concentration profiles corresponding to the density plots shown in Fig. 5 at (a) constant injection U=0.001m/s (b) T=50 (c) T=100. The arrow shows the increase in time. The time stamps are same as in Fig. 5.

First, the effect of Γ and T is presented when the sample is more viscous than the surrounding fluid, i.e., R>0. Fig. 7, Fig. 8 represents $c_{avg}\left(x,t\right)$ corresponding to the simulations of Fig. 3, Fig. 5 for injection-extraction and extraction-injection process for R=2. In both Fig. 7, Fig. 8, the average concentration profile of the sample shows distortions due to the VF on the rear interface.

The appearance of bumps at the corresponding interface reveals the existence of fingers at the unstable interface, while the stable interface is characterized by simple dispersion. The interaction between both interfaces is witnessed by the passage of time, a feature unique to the VF of a finite sample. Eventually, the maximum concentration decreases as a consequence of dispersion and fingering of the sample. Viscous fingering shifts the mean position of the sample while also distorting and widening the peak [3]. For Γ=2, the sample moves downstream very quickly in comparison to Γ=−2, for higher values of T. In particular, for time-period T=100 and amplitude Γ=−2, it is observed that the sample moves backwards in the upstream direction and the diffusive interface intact its profile. This effect observed due to the fact that for the half cycle of the injection U(t)<U_0 for Γ=−2. It is further noticed that with increase in time-period, the widening of sample and peak distortions are prominent for injection-extraction process.

**Fig. 9:**
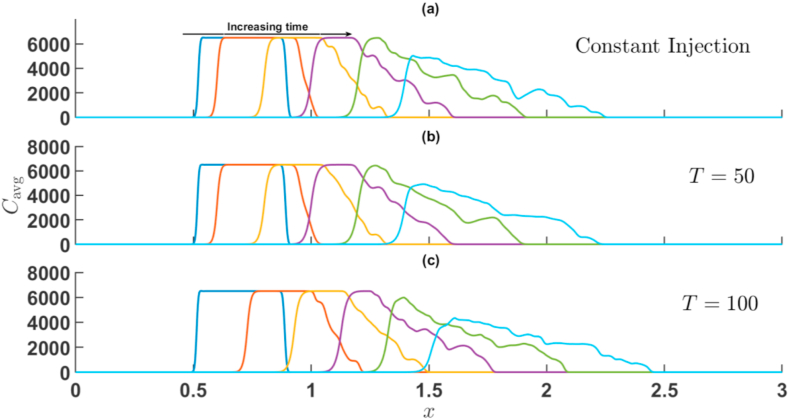
For Γ=2,R=−2 the transversely averaged concentration profiles corresponding to the dynamics shown in Fig. 4 at (a) constant injection U=0.001m/s (b) T=50 (c) T=100. The arrow shows the increase in time. The time stamps are same as in Fig. 4.

**Fig. 10:**
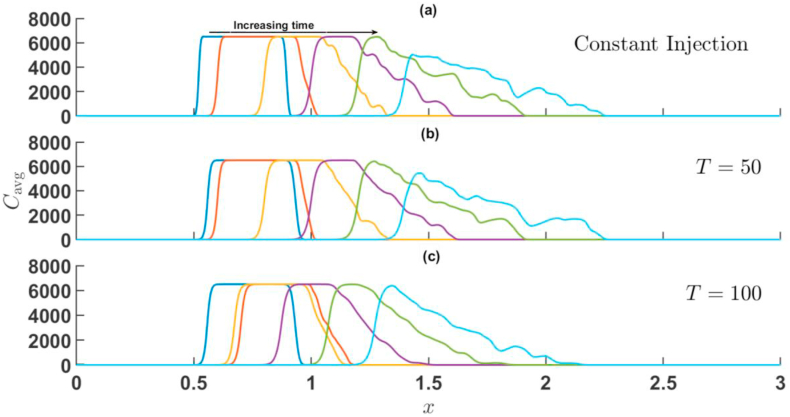
For Γ=−2,R=−2 the transversely averaged concentration profiles corresponding to the dynamics shown in Fig. 6 at (a) constant injection U=0.001m/s (b) T=50 (c) T=100. The onset gets delayed with increase in T. The arrow shows the increase in time. The time stamps are same as in Fig. 6.

When the sample is less viscous than the surrounding fluid, i.e., R<0. For R=−2 the average concentration field is depicted in Fig. 9, Fig. 10 corresponding to the simulations of Fig. 4, Fig. 6 for injection-extraction and extraction-injection process with R<0. In this case, the frontal interface experiences an unfavourable viscosity contrast and the rare interface followed a diffusive profile. In case of injection-extraction case, the breakthrough of fingers set early with increase in T whereas for the extraction-injection scenario, the delayed onset is observed (see Fig. 10). The onset time for Γ=−2 is 10 s, 38 s, and 68 s for U=0.001m/s, T=50, and T=100, respectively. Further, similar to R>0, the backward movement of concentration profile can be seen at t=150,T=100 and Γ=−2. For Γ=−2, the distortion of the peaks decreases with increase in the value of time-period T and the sample distortion is maximum in case of constant injection in comparison to its time-dependent counter parts. Thus, in chemical separation technique, when the sample is less viscous, the extraction-injection strategy is very useful. In order to quantify further effects of Γ and T, a statistical quantification based on variance of the concentration is presented in the next section.

### 4.3 Statistical quantification

**Fig. 11:**
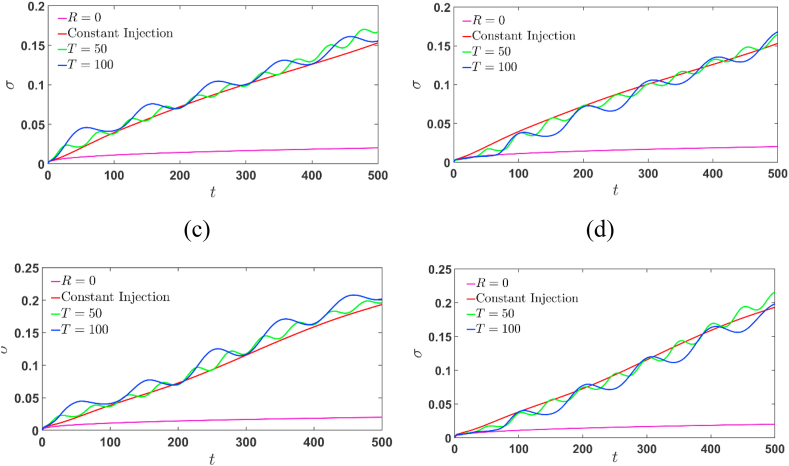
The oscillatory behaviour of standard deviation for injection-extraction process with Γ=2 is shown in (a) and (c), and that of extraction-injection with Γ=−2 is illustrated in (b) and (d). The other parameters chosen are R=2,R−2,T=50,100.

To quantify the effects of viscous fingering on peak broadening and distortion of the sample, when the sample is less or more viscous, we compute the variance of the transversely averaged concentration profiles which indicates the width of the distribution of concentration [3,7] as

(14) $$\sigma^{2}\left(t\right)=\frac{\int_{0}^{L_{x}}c_{avg}\left(x,t\right){\left[\left(x-m\left(t\right)\right)\right]}^{2}dx}{\int_{0}^{L_{x}}c_{avg}\left(x,t\right)dx}\text{,}$$

where, $m\left(t\right)=\int_{0}^{L_{x}}x\;f\left(x,t\right)\;dx\text{,}$ and $f\left(x,t\right)=\frac{c_{avg}\left(x,t\right)}{\int_{0}^{L_{x}}c_{avg}\left(x,t\right)dx}$,

It must be noted that Eq. (14) is the first statistical moment and f(x,t) is the probability density function of the continuous distribution $c_{avg}\left(x,t\right)$. The contribution of viscous fingering to the variance $\sigma^{2}$ is the quantity $\sigma_{f}=\sqrt{\sigma^{2}-\sigma_{0}^{2}}$. Here $\sigma_{0}^{2}=\frac{l^{2}}{12}+2t$ is the variance of pure diffusion (*R* = 0), where the first term is due to the initial sample width and the second term is due to dispersive mixing [7]. As noted by De Wit et al. [7] in VF for finite sample the variance of the sample is enhanced due to fingering for the sample being more or less viscous than the pure diffusion case. Fig. 11 (a) and (c) represents the standard deviation σ of the concentration for injection-extraction case, while Fig. 11 (b) and (d) depicts the extraction-injection case. With the onset of fingering σ deviates from that of pure diffusion case, $\sigma_{0}$ and gets more pronounced with the increase in time. It is expected that σ eventually approaches an asymptotic value when the unstable interface interacts with stable interface. For Γ=2, σ is always larger than the constant injection case and it deviates earlier from $\sigma_{0}$ than Γ=−2. This is attributed to the fact that for the initial period the diffusion is dominant in the extraction-injection case and hence the delay in onset of fingers is observed. Further, for fixed R the onset is early for Γ=2 and it gets delayed for Γ=−2, with increase in T. This confirms our assertion that the finite sample, the extraction-injection can be preferred over injection-extraction process. It would be encouraging to see how the cyclic injection alter the other hydrodynamic stability behaviour of fingers which is addressed in the next section.

## 5 Quantification of stability

Various characteristics have been employed to study the quantitative characterisation of the instability generated by the sinusoidal injection velocity. Interfacial length, degree of mixing, and mixing length, in particular, are used to assess the effect of time-dependent velocity on flow dynamics. These metrics aid in assessing hydrodynamic instability by capturing finger formation, fingering zone length, and degree of instability.

### 5.1 Interfacial length

The emergence of fingering instability and the interactions between stable and unstable interfaces can be quantified adequately using the interfacial length's temporal development, which is defined as shown in Eq. (15) [3].

(15) IL(t)=∫0Lx∫0Ly[(∂c∂x)2+(∂c∂y)2]12dxdy

**Fig. 12:**
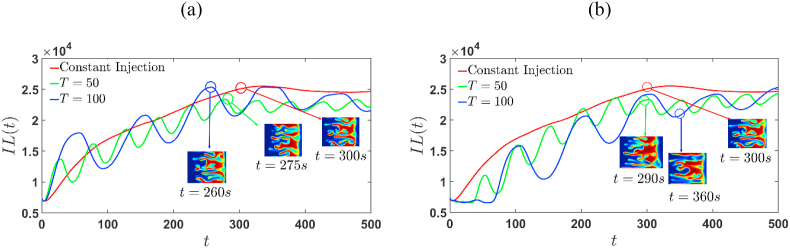
Temporal evolution of the interfacial length for the simulations of R=2, (a) Γ=2, and (b) Γ=−2, shown in Fig. 3, Fig. 5. IL (t) increases with increasing T for Γ=2 whereas for Γ=−2 it decreases for initial times and gradually stabilizes at later times with increasing T. For Γ=-2 , the IL (t) is constant during the initial diffusive regime, whereas it increases strongly for Γ=2. The concentration distribution is depicted in the inset images approximately at the time when stable and unstable interfaces interact.

**Fig. 13:**
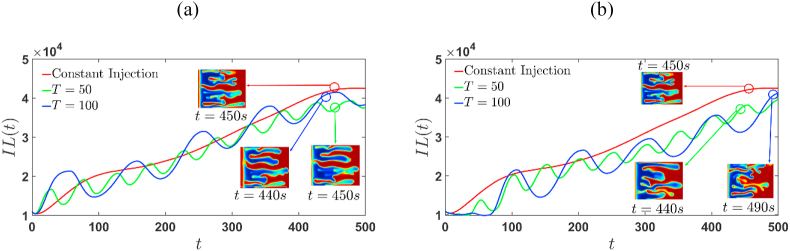
Temporal evolution of the interfacial length for the simulations of R=−2, (a) Γ=2, and (b) Γ=−2, shown in Fig. 4, Fig. 6. For Γ=−2, IL (t) is constant during the initial diffusive regime, whereas it increases strongly for Γ=2. The concentration distribution is depicted in the inset images approximately at the time when stable and unstable interfaces interact.

The interfacial length also referred as relative contact area, is a measurement of the temporal evolution of transverse and axial concentration gradients. The variation of IL(t) is illustrated in Fig. 12, Fig. 13 for positive and negative R, respectively. In the diffusion regime, IL(t) remains constant and increases as the fingers develop. As a result, the commencement of fingering is indicated when IL(t) begins to increase from its constant value in the diffusive regime. The inset images in Fig. 12, Fig. 13 reflect the concentration distributions approximately at the time of interface interaction. In case of sinusoidal time-dependent injection, IL(t) increases prominently in the first half of the cycle and decreases rapidly during the next half. These trends are in contrast with those of the constant injection case, in which the IL(t) monotonically increases after the onset of instability until the unstable interface interact with stable interface, as shown in Fig. 12, Fig. 13. When the sample is more viscous, the temporal evolution of the interfacial length corresponds to the simulations of extraction-injection (Γ=−2), the displacements remain stable until t∼30s and t∼65s for T=50 and T=100, respectively. In Table 3 and Table 4, the time of onset of fingers and stable regions is summarized. Given an injection strategy, the onset time for fingering remains for both R>0 and R<0, since the stable interface acts as barrier for the growth of formed fingers.

**Table 3:** Diffusive region and onset of fingers for T=50. The onset remains almost same independent of the log-mobility ratio.

	Constant Injection		Γ=2		Γ=−2	
	R=2	R=−2	R=2	R=−2	R=2	R=−2
Diffusive time interval	[0,4]	[0,9]	[0,2]	[0,4]	[0,34]	[0,37]
Onset	5s	10s	3s	5s	35s	38s

**Table 4:** Diffusive region and onset of fingers for T=100. The onset remains almost same independent of the log-mobility ratio.

	Constant Injection		Γ=2		Γ=−2	
	R=2	R=−2	R=2	R=−2	R=2	R=−2
Diffusive time interval	[0,4]	[0,9]	[0,2]	[0,4]	[0,67]	[0,67]
Onset	5s	10s	3s	5s	68s	68s

Inset images depict the concentration distributions at the time of interaction and how this time differs in the time-injection procedure and the continuous injection process. The qualitative structure of IL(t) remains same, while quantitative measurements alter owing to the injection-extraction and extraction-injection processes. In particular, for any value of R, when Γ=−2, IL(t) is always lower than the constant case and is almost same for Γ=2. In addition, it is observed that under time-dependent strategies, with increasing time-period, the splitting of fingers is observed because of steeper concentration gradients at the unstable front, which is due to the stretching associated with a stronger cross flow [14]. It should be noted that after the finger split, they become narrower and longer as they become more stretched (see Figs. 3–6). In the case of extraction-injection process, $U\left(t\right)<U_{0}$ in the first half-cycle which results in dominant diffusive forces over convective forces as observed in Figs. 12 (b) and Fig. 13 (b). Thus. For any R and Γ=−2, the increase in time-period T delays in onset of fingers. Further, at later period the convective forces overcomes the diffusive forces with the onset of finger and hence IL(t) grows stronger.

In the case of injection-extraction process (see Figs. 12 (a) and Fig. 13 (a)) convection dominates over diffusion and strong growth of IL (t) from an early time, indicating an early onset of fingers. Furthermore, increasing the time period T has no effect on the onset of fingers, and the interaction of the front and rear interfaces sets early. Therefore, it can be established that the extraction-injection process is a better option when the less viscous sample is surrounded by a more viscous fluid. By prolonging the fluid-fluid interface, VF modifies the concentration gradient and the area available for diffusive flux across the interface and thus modifies the rate of mixing. It would be interesting to investigate the impact of cyclic injection on the mixing of a viscous finite slice.

### 5.2 Degree of mixing

The degree of mixing characterizes the mixing of the finite sample with the surrounding fluid, represented as given in Eq. (16):

(16) $$\chi \left(t\right)=1-\frac{\sigma^{2}\left(t\right)}{\sigma_{\max}^{2}}$$

defined in terms of the global variance $\sigma =\left\langle C^{2}\right\rangle -{\left\langle C\right\rangle }^{2}$ of the concentration field where ⟨⋅⟩ denotes spatial averaging over the domain volume [26]. The maximum variance, $\sigma_{\max}^{2}$ represents a perfectly segregated state and $\sigma^{2}$ = 0 and χ = 1 corresponds to a perfectly mixed state [26]. It is observed that $\lim_{t\to 0}\chi \left(t\right)=0$, as at the initial time the interface is assumed to be sharp. As time evolves the value of χ (t) increases as the two fluids begin to interact, resulting in the development of fingering dynamics.

**Fig. 14:**
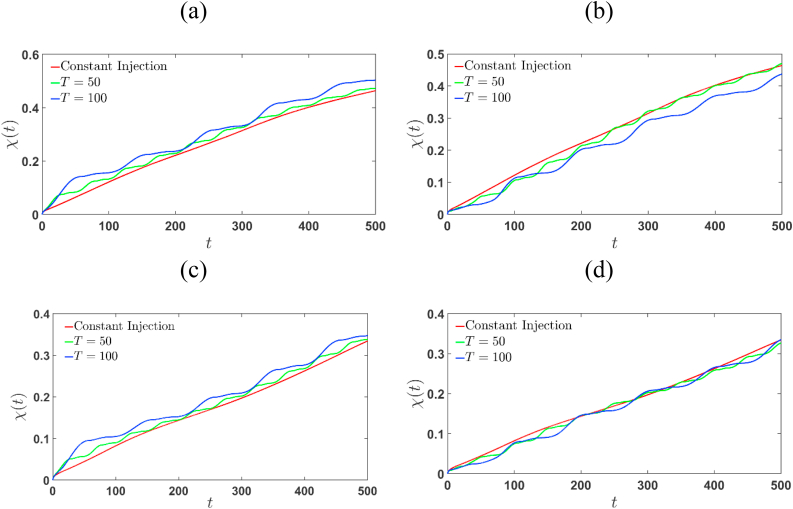
Temporal evolution of the degree of mixing for R=2, (a) Γ=2, and (b) Γ=−2, corresponding the surface plots depicted in Fig. 3, Fig. 5, respectively. The degree of mixing for R=−2 associated to Fig. 4, Fig. 6 is presented in (c) and (d), respectively. The χ(t) enhances with increasing values of T for Γ=2 whereas it decreases for Γ=−2.

The degree of mixing for the injection-extraction process (Γ>1) is always remain higher than the constant one (see Fig. 14 (a) and (c)), while for the extraction-injection (Γ<1) scenario the mixing degree remains lower than the constant injection (see Fig. 14 (b) and (d)), irrespective of whether the sample is more or less viscous. This indicates that in case of Γ=2, mixing is prominent for both R>0 and R<0. In particular, the mixing length of constant injection is indistinguishable for R=−2 and Γ=−2. Hence in the injection-extraction process mixing is profound which is expected as the onset injection velocity is higher than other injection strategy mentioned in the current study. This confirm our observation on interfacial length where it is noted that for Γ=2, the onset and fingering interaction is most vigour which results is optimal mixing.

**Fig. 15:**
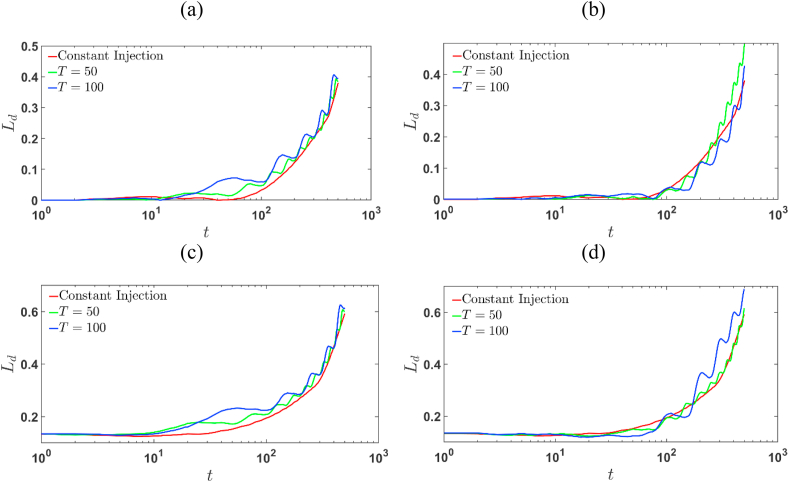
The semi-log plot (log scale in t axis) of the mixing length for R=2, (a) Γ=2, and (b) Γ=−2, corresponding to the concentration plots shown in Fig. 3, Fig. 5, respectively. The mixing length related to the simulations shown in Fig. 4, Fig. 6 of R=−2, are shown in (c) and (d) respectively. For given T and R, the mixing length is more for Γ=2 in comparison to Γ=−2.

### 5.3 Mixing length

The mixing length is another classical measure for the quantitative analysis of the VF which determines the extent of the zone where the two miscible fluids mix one into the other [1]. The mixing length $\left(L_{d}\right)$ can be defined as the length of the interval between the pure diffusive case i.e., R=0 and R≠0 in which the $c_{avg}>0.001$.

The semi-log plot of mixing length, $L_{d}$ is illustrated in Fig. 15 (a)–(d) for the constant and time-dependent injections with parameters T=50 and 100 and Γ=2 and −2. For fixed R and T, the mixing length, $L_{d}$ is larger in the injection-extraction process than constant and the extraction injection process. In case T fixed, the finger spread most for R=2 and Γ=2. Overall, it is observed that in the case of injection-extraction process the mixing length is always more than that of the constant injection. This substantiate our assertion that the spreading of the sample is more in case of injection-extraction process independent of the value of log-mobility ratio. In case of extraction-injection process with R=−2, $L_{d}$ is least among the rest of the injection strategy and almost identical to the constant injection at the later time.

### 5.4 Average convective and diffusive forces

In Eq. (4), the term $\vec{u}\cdot \nabla c$ describes the convection and ∇⋅(D∇c) represents the diffusive force. Further understanding on the effect of Γ and T on the fingering dynamics may be obtained by analyzing the contribution of these diffusive and convective terms. The absolute forces for these two terms are defined as [27]:

(17) $$C_{f}=\int_{0}^{Lx}\int_{0}^{Ly}|\vec{u}∙\nabla c|\;dxdy$$

(18) $$D_{f}=\int_{0}^{Lx}\int_{0}^{Ly}\left|\nabla ∙\left(D\nabla c\right)\right|\;dxdy$$

**Fig. 16:**
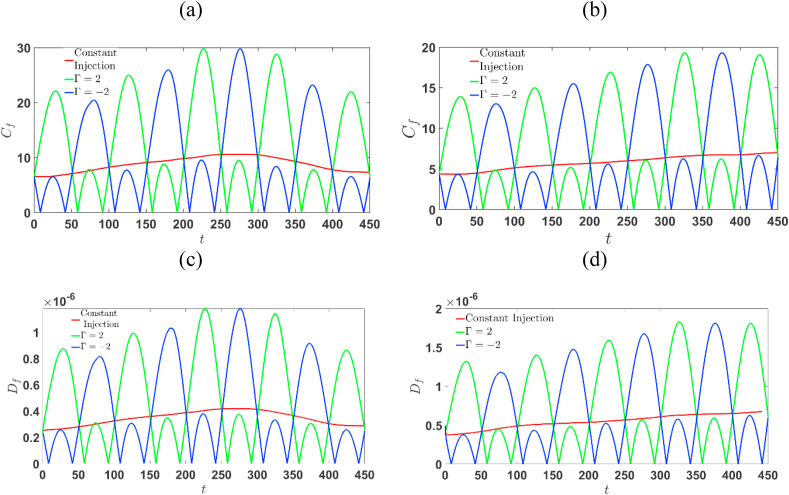
For T=100, (a) and (c) depicts the absolute average $C_{f}$ and $D_{f}$ for R>0; (b) and (d) depicts the absolute average $C_{f}$ and $D_{f}$ for R<0.

In particular $C_{f}$ and $D_{f}$ represented by Eqs. (17), (18) quantify and compare the relative contributions of convection and diffusion terms for time-dependent injection and constant injection. The general evolution of these terms is associated with the development and nonlinear interactions of the fingers. Fig. 16 (a)-(d) depicts the variation of convective and diffusive forces with time for both R>0 and R<0 for a fixed time-period T=100. Except possibly at early times, convective forces are generally greater than diffusive forces. However, in the extraction-injection case (Γ<−1), the opposite behaviour is observed, as shown in Fig. 16, which is independent of the sign of R. This is due to the fact that in the extraction-injection case for half cycle $U\left(t\right)<U_{0}$ which makes the diffusive forces to be more prominent. One striking feature of time-dependent case is that the convective, $C_{f}$ forces and diffusive forces $D_{f}$ are non-monotone, in particular these are cyclic. Further, in any particular time window, whenever $D_{f}$ is weaken, $C_{f}$ is dominant and these process goes in a cyclic manner. For Γ>1, the convective forces are most dominant in comparison to other cases when $U\left(t\right)>U_{0}$. In time-dependent case, both $D_{f}$ and $C_{f}$ are non-monotonically increasing for a longer period when the finite slice is less viscous, that is R<0, than R>0.

This can be explained as follows: while the front interface remains stable, the back interface develops fingers, causing the sample's center of gravity to shift backwards over time in relation to its initial position. This dynamic arises from the fact that the stable zone acts as a barrier to finger propagation in the flow direction, resulting in reverse fingering. Further the diffusive and convective forces for constant injection is the mean of the injection-extraction and extraction-injection case. This reflects that throughout the flow process the same amount of fluid is injected whether the injection velocity U is constant or cyclic. In particular, for Γ=−2,R=−2 (see Fig. 16 (b) and (d)), the diffusive forces are most dominant. This is in-lined with the previous analysis of degree of mixing and interfacial length, where its is shown that the finger development and mixing is minimum for Γ=−2,R=−2 . As the diffusive forces are more dominant, the onset of fingers are set at a very late time (t≈68s) which is shown in Fig. 6 (c). Thus, the interplay of $D_{f}$ and $C_{f}$ suggest that if the sample is less viscous then the extraction-injection strategy is useful.

## 6 Conclusion

In a finite region, the displaced fluid in a chromatographic column or subsurface aquifers remains connected. The finite sample is a rectangular slice that is surrounded by the displacing fluid in both the upstream and downstream directions. When the viscosity of the localized fluid is less (more) than that of the ambient fluid, the frontal (rear) interface becomes unstable. This is represented by <0 (R>0) in an Arrhenius type viscosity-concentration relation stated in Eq. (5). The non-linear simulations have been carried out based on a finite element method using COMSOL Multiphysics, to determine the effects of two parameters characterizing sinusoidal time-dependent displacements, namely, the period T and the amplitude Γ. The development of instabilities at two interfaces with time was simulated in a such a way that the numerical dispersion is minimized and mesh refinement is optimized.

More importantly, the unstable displacement and sweep efficiency were studied using a time-dependent injection rate involving periodic injection and extraction alternation. Unlike the commonly used constant injection rate, this time-dependent displacement rate resulted in different flow dynamics and sweep efficiency, despite the same amount of fluid being injected. Although Yuan and Azeiz [15,16,27,28] and Elgahaway and Azeiz [14] thoroughly investigated the effect of a cycle period for single interface, there is no study available for the finite sample. In a finite miscible sample, it has been demonstrated that for Γ>1, longer periods result in stronger instabilities with longer, more developed fingers and Γ<−1, results in a less unstable displacement with fewer fingers and a more diffused front. In the case of a single interface, the reverse dynamics were observed for sinusoidal injection velocity. The analysis of the interfacial length (IL), degree of mixing, and variance confirms these qualitative trends. A longer period results in a higher IL for Γ>1 and a lower IL for Γ<−1. It is also worth noting that longer periods result in a longer breakthrough time for Γ<−1 but have little effect on the breakthrough time for Γ>1. Moreover, the displacements with sinusoidal injection with Γ<−1 (extraction-injection) are more stable than their counterparts with constant injection and Γ>1, regardless of the sign of the log-mobility R.

This study demonstrated conclusively that it is possible to alter or control the instability in a miscible slice by selecting the two parameters that characterize the time-dependent flow, namely the period T and the amplitude Γ. For a fixed R the displacement process initiating the flow by extraction vs injection plays a critical role in flow development, and the period T determines the nature of the finger structures as well as other important flow characteristics such as the breakthrough time. This suggests that the extraction-injection process attenuates finger development, whereas the injection-extraction process is conducive for mixing. The implications of these findings are more nuanced than they appear at first glance. In addition, the results of the current work indicate that the extraction-injection method is beneficial for chemical separation, while the injection-extraction process will be advantageous for fluid mixing. It is expected that the sinusoidal injection process has the potential to alter the miscible flooding in other physical processes such as reactive fluids [29] and enhanced oil recovery [30], to name a few.

## Author contribution statement

Syed Zahid: Performed the experiments; Analyzed and interpreted the data; Contributed reagents, materials, analysis tools or data; Wrote the paper.

Surfarazhussain S. Halkarni: Conceived and designed the experiments; Analyzed and interpreted the data.

Tapan Kumar Hota: Conceived and designed the experiments; Analyzed and interpreted the data; Contributed reagents, materials, analysis tools or data; Wrote the paper.

## Funding statement

This work was supported partially by SRM University *AP,* and Science and Engineering Research Board (SERB), Department of Science and Technology, Government of India (Grant No. SRG/2020/000713).

## Data availability statement

Data will be made available on request.

## Declaration of interest’s statement

The authors declare no conflict of interest.
